# Editorial for the Special Issue on Advances in Digital Manufacturing and Nano Fabrication

**DOI:** 10.3390/mi17091057

**Published:** 2026-09-04

**Authors:** Jian Cheng, Jianguo Zhang, Qi Liu

**Affiliations:** 1 State Key Laboratory of Robotics and System, School of Mechatronics Engineering, Harbin Institute of Technology, Harbin 150001, China; 2 School of Mechanical Science & Engineering, Huazhong University of Science and Technology, Wuhan 430074, China; zhangjg@hust.edu.cn; 3 Department of Mechanical Engineering, University of Bath, Bath BA2 7AY, UK; ql787@bath.ac.uk

In recent years, digital manufacturing, high-energy-beam processing, and nano- and micro-scale fabrication have developed rapidly, extending the capabilities of modern engineering and broadening their practical application [1,2]. At the same time, these advances have increased the demands placed on both material systems and manufacturing methods [3,4]. The central challenge is no longer only developing advanced materials but also establishing suitable process chains that allow their geometric, structural, optical, and functional performance to be realized reliably [5,6]. In this context, advanced manufacturing has evolved from material shaping into an integrated field encompassing digital design, process innovation, precision fabrication, structural and functional integration, and intelligent production systems [7].

To fully exploit advanced materials and components, research has therefore focused on high-resolution fabrication, novel manufacturing methodologies, post-processing, and auxiliary characterization [8,9]. These stages determine final-quality metrics such as dimensional accuracy, surface integrity, defect population, residual stress, optical loss, laser-damage resistance, fatigue performance, wear resistance, and service reliability [10,11]. This dependence is especially pronounced in high-energy-beam, additive, thin-film deposition, and ultra-precision processes, for which as-fabricated components often require machining, polishing, heat treatment, ion-beam regulation, or other surface modification steps before meeting engineering specifications [12].

Meanwhile, modeling and numerical analysis have become indispensable for understanding the fundamental mechanisms of advanced manufacturing processes and optimizing process parameters [5,6]. Multiphysics simulations and data-driven surrogate models are increasingly used to examine particle transport, heat and mass transfer, stress evolution, microstructure development, defect formation, optical performance, and material removal in laser processing, additive manufacturing, thin-film deposition, and micro/nano fabrication [7,8,10]. These methods are particularly valuable because many critical phenomena occur in highly transient and localized environments that are difficult to observe directly [9,11]. Simulation- and model-assisted process analysis can therefore reduce trial-and-error experimentation, clarify competing performance objectives, improve process reliability, and accelerate technological development [7,12].

In parallel, advanced detection, monitoring, and intelligent control systems are re-shaping modern manufacturing [1,2]. In situ sensing, defect detection, and photoluminescence-based characterization [8,9], together with machine learning and physics-informed control [3,7], are enabling a transition from empirical parameter adjustment to data-driven, closed-loop, and self-optimized operation. By connecting process conditions with surface morphology, atomic-defect states, optical properties, and residual stress [8,10], these systems can improve consistency and quality while supporting the scalable manufacture of high-performance components [1,2].

This Special Issue, “Advances in Digital Manufacturing and Nano Fabrication,” collects twelve contributions, comprising eleven original research articles and one review. These papers encompass five major themes: (1) data-driven inspection and intelligent process optimization; (2) integrated digital design and precision manufacturing; (3) laser-enabled micro/nano fabrication and surface engineering; (4) advanced subtractive processing and component repair; and (5) directed energy deposition and process-property control. Collectively, the contributions deepen our understanding of state-of-the-art manufacturing technologies and provide a scientific and technological basis for more-intelligent, reliable, and application-oriented production. The principal findings and scientific significance of the included articles are summarized below.

Operating in the field of digital manufacturing and intelligent process optimization, Gao et al. [Contribution 1] developed a lightweight Ghost-BiFPN-Efficient-YOLOv8 model for detecting surface defects on printed circuit boards. The model achieved a mean average precision of 98.9% at an intersection-over-union threshold of 0.5 while maintaining 2.6 million parameters and an inference speed of 252 frames per second, demonstrating a balance between detection accuracy and computational efficiency. Wei et al. [Contribution 2] combined deposition experiments, surface characterization, mechanism analysis, and Bayesian optimization to predict the refractive index and surface roughness of Ta_2_O_5_ films produced via ion-assisted electron beam evaporation. Their analysis identifies ion-source beam voltage and oxygen flow rate as the principal variables governing deposition quality and provides preferred parameter ranges for process design. Churchill-Baird et al. [Contribution 3] established an integrated CAD/CAM framework for the parametric design and multi-axis single-point diamond cutting of planar curvilinear V-grooves. The framework was validated via fabrication of sinusoidal grooves and a functional capillary-flow pattern. Tan et al. [Contribution 4] integrated grey relational analysis, the technique for order preference by similarity to an ideal solution, and random forest regression to optimize burr formation and surface quality in the micro-milling of Ti-6Al-4V alloy. The resulting model captured nonlinear relationships between machining parameters and outcomes and improved prediction accuracy relative to conventional linear regression.

Several contributions focus on laser-enabled processing and the control of optical or functional surfaces. Shen et al. [Contribution 5] developed a coupled photoelectric–thermal stress model to investigate laser-induced damage initiated by extrinsic defects in wavelength-separation coatings. Their results clarify how defect location, type, and size influence local optical-field enhancement, temperature, and thermal stress, providing a basis for defining defect-control requirements in coating manufacture. Tan et al. [Contribution 6] experimentally optimized CO_2_ laser polishing of fused silica and reported an approximately 20% increase in the laser-induced damage threshold, along with a 76.4–90.8% reduction in damage density relative conventionally polished and etched samples. Zhuo et al. [Contribution 7] examined surface-profile formation during continuous-wave CO_2_ laser processing. Their multivariate regression model predicted profile errors with less than 5% deviation, and the introduction of an annealing step reduced the profile error by more than 50%. Lin et al. [Contribution 8] investigated the femtosecond-laser precision etching of a silver layer deposited on silica-fiber-reinforced silica aerogel. Through orthogonal experiments and compositional analyses, they optimized the etching parameters and showed that the material removal process was governed primarily by a photothermal mechanism. Wang et al. [Contribution 9] studied rectangular laser-textured patterns on silicon nitride ceramic and established relationships between scanning conditions, groove geometry, surface chemistry, and wettability. The laser treatment changed the water contact angle from 35.51 ± 0.33° to as high as 57.52 ± 1.83°, demonstrating controllable modification of surface wetting behavior. Liu et al. [Contribution 10] reviewed recent progress in laser-assisted precision machining, covering laser-assisted cutting, grinding, milling, drilling, and polishing. Their review summarizes the mechanisms by which laser energy modifies the thermomechanical response and machinability of difficult-to-machine materials and outlines future directions for process integration and control.

The Special Issue also includes studies on advanced subtractive and additive manufacturing. Xia et al. [Contribution 11] investigated directed energy deposition of Hastelloy X cladding on a 50CrVA substrate for the repair of aircraft landing gear. By combining thermal-flow simulation with orthogonal experiments, they optimized laser power, powder feed rate, scanning speed, and overlap rate, producing cladding with improved corrosion resistance while retaining wear resistance comparable to that of the substrate. Wang et al. [Contribution 12] introduced elliptical vibration micro-turning for producing short, spiral-shaped silk-fibroin chips with potential use as drug-delivery carriers. A coupled finite-element and smoothed-particle-hydrodynamics model, supported by experiments, showed how vibration frequency and amplitude regulate chip curling, breakage, and cutting force.

Taken together, these contributions illustrate how computational approaches and advanced physical processes are converging across the manufacturing chain, with nano fabrication providing an increasingly important route to the precise control of structures, surfaces, and defects. Laser-based, thin-film, vibration-assisted, and additive processes offer increasingly precise control over material removal, surface functionality, defect formation, and component repair, including at the micro- and nanometer scales. Modeling, data-driven optimization, in situ sensing, and closed-loop control complement these processes by linking fabrication parameters to material structure and component performance. Future progress in nano fabrication will depend on higher spatial resolution, tighter control of defects and surface integrity, and scalable process integration, alongside improved energy efficiency and process robustness. Through this Special Issue, we hope to provide researchers and practitioners with a concise view of recent advances in digital manufacturing and nano fabrication and stimulate further work toward intelligent, reliable, and application-oriented manufacturing systems.
